# Chemical Recycling of Poly(Cyclohexene Carbonate) Using a Di‐Mg^II^ Catalyst

**DOI:** 10.1002/anie.202201785

**Published:** 2022-05-05

**Authors:** Frances N. Singer, Arron C. Deacy, Thomas M. McGuire, Charlotte K. Williams, Antoine Buchard

**Affiliations:** ^1^ Department of Chemistry University of Bath Centre for Sustainable and Circular Technologies Claverton Down, Bath BA2 7AY UK; ^2^ Department of Chemistry University of Oxford Chemistry Research Laboratory 12 Mansfield Rd Oxford OX1 3TA UK

**Keywords:** Carbon Dioxide, Catalysis, Depolymerization, Epoxide, Recycling

## Abstract

Chemical recycling of polymers to true monomers is pivotal for a circular plastics economy. Here, the first catalyzed chemical recycling of the widely investigated carbon dioxide derived polymer, poly(cyclohexene carbonate), to cyclohexene oxide and carbon dioxide is reported. The reaction requires dinuclear catalysis, with the di‐Mg^II^ catalyst showing both high monomer selectivity (>98 %) and activity (TOF=150 h^−1^, 0.33 mol %, 120 °C). The depolymerization occurs via a chain‐end catalyzed depolymerization mechanism and DFT calculations indicate the high selectivity arises from Mg‐alkoxide catalyzed epoxide extrusion being kinetically favorable compared to cyclic carbonate formation.

## Graphical abstract

Here, the chemical recycling of poly(cyclohexene carbonate) (PCHC) to cyclohexene oxide (CHO) with 98 % selectivity using a dinuclear Mg^II^ catalyst is reported. The chemical recycling and recovery of CHO under mild conditions further advances the utilization of CO_2_‐polycarbonates as recyclable materials.

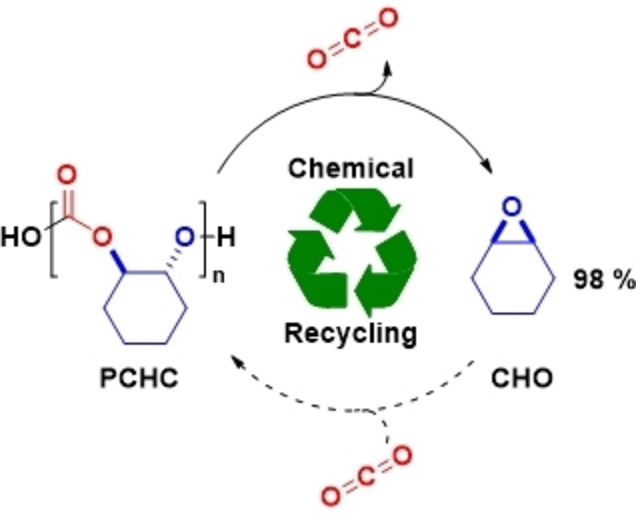

Polymer chemical recycling to re‐form the constituent monomers, and their subsequent re‐polymerization, is an attractive future waste management solution.^[1]^ After re‐polymerization the polymer properties are uncompromised, the process should operate over multiple cycles, and this type of chemical recycling should also reduce, and may even eliminate, the need for virgin petrochemicals.^[1]^ In the case of epoxides, such recycling would be expected to result in significant energy savings as well as other environmental impact reductions.^[2]^ Chemical recycling processes are already scalable for polyesters, e.g. glycolysis of PET,^[3]^ and other oxygenated polymers.[^1a^, ^1b^, ^1c^, ^1d^] Polymerizations which have low‐exergonicity (i.e. close to equilibrium) are best suited to selective chemical recycling as they have minimized energy requirements.^[1e]^ Recently the chemical recycling of various new polyethers,^[1b]^ ‐esters[^1c^, ^1d^, ^4^] and ‐carbonates made by ring‐opening polymerization has been demonstrated.[^1e^, ^5^] For example, Coates and team recycled poly(1,3‐dioxolane) to monomer in high yield (98 %).^[1b]^ Chemical recycling of some aliphatic polycarbonates to various cyclic carbonates is also known, and is managed by manipulating the monomer‐polymer equilibria, i.e. by catalyzed back‐reactions above the polymer's ceiling temperature, *T*_c_.^[6]^ One challenge of exploiting such polymer–monomer equilibria is that the most “recyclable” polymers tend to be the least successfully synthesized (equilibria lie towards monomers) and may have undesirably low thermal stability (low ceiling temperatures). Another issue is that ring‐opening polymerization is more problematic using heterocycles incorporating aromatic, rigid or functional substituents, thereby limiting the properties for the “recyclable” polymers.[^1c^, ^1d^, ^7^]

Epoxide/carbon dioxide ring‐opening copolymerization (ROCOP) is an attractive route to polycarbonates.^[8]^ Since the polymerization is driven by the opening of the high ring‐strain epoxide, it achieves high conversions and produces many different polycarbonates.^[8c]^ Although the thermodynamic product of epoxide/carbon dioxide coupling is a 5‐membered cyclic carbonate (CHC), judicious selection of catalysts and conditions delivers high polymer selectivity even at high temperatures and using neat monomer.^[8]^ Nonetheless, the chemical recycling of carbon dioxide derived polymers is challenged by the cyclic carbonate stability.^[9]^ True chemical recycling requires polycarbonate depolymerization to epoxides and carbon dioxide. Some special epoxides are amenable to chemical recycling, most notably cyclopentene oxide (CPO) or limonene oxide (LO),[^5a^, ^9^, ^10^] but until now effective chemical recycling of the most widely investigated poly(cyclohexene carbonate) PCHC has not been possible (Scheme S1).

In 2013, Darensbourg and co‐workers reported the first polycarbonate chemical recycling by reacting poly(cyclopentene carbonate) (PCPC) with a [(salen)Cr^III^Cl]/tetrabutylammonium azide (^n^Bu_4_NN_3_) catalyst system to yield 92 % cyclopentene oxide and 8 % cyclic carbonate (2 mol % catalyst, 110 °C, toluene, 30 h).^[10a]^ Later, poly(indene carbonate) was also depolymerized but with low selectivity for indene oxide (9 %).^[11]^ In 2017, Lu and co‐workers depolymerized poly(BEP carbonate) (BEP=benzyloxycarbonyl‐3,4‐epoxy pyrrolidine) with >99 % epoxide selectivity, using a [(salen)Cr^III^Cl]_2_/ 2 PPNX (X=Cl, F, NO_3_^−^ or N_3_^−^) catalyst system.^[5a]^ Subsequently, successful polymerization/ depolymerization cycles were demonstrated using a series of BEP epoxides with different N‐protecting groups.^[12]^ In 2017, Koning and team reported the depolymerization of poly(limonene carbonate) (PLC) to limonene oxide, with >99 % selectivity, using a 1,5,7‐triazabicycle[4.4.0]dec‐5‐ene (TBD) catalyst (4 mol % catalyst loading, 110 °C, toluene).^[10b]^ Subsequently, poly(limonene carbonate) depolymerization as part of an ABA‐triblock polymer was reported using a dizinc catalyst.^[13]^ Although selective polycarbonate chemical recycling is feasible,[^10a^, ^10b^] these CO_2_‐derived copolymers (PCPC, PLC, P(BEPC)) are not so widely investigated.

Most CO_2_/epoxide ROCOP investigations apply poly(cyclohexene carbonate) (PCHC) as the work‐horse polymer and it is used for almost all catalyst comparisons.^[8]^ PCHC has an attractive high glass transition temperature and shows high tensile strength — it could be a useful sustainable engineering plastic.^[14]^ PCHC ductility and toughness can be improved by block polymer formation and such materials show promise as adhesives, elastomers or as toughened plastics.^[14]^ One limitation is the energy cost of CHO production — either by sourcing it from benzene or using recently reported routes from fatty acids.^[8b]^ To date, there are only investigations of poly(cyclohexene carbonate) decomposition to cyclic carbonate (cyclohexene carbonate, CHC).[^5b^, ^9^, ^15^] PCHC recycling to cyclohexene oxide (CHO) might be feasible if the barrier to epoxide formation were reduced. Prior DFT investigations of di‐Zn^II^ ROCOP catalyst, active at low CO_2_ pressures (1 bar), showed low barriers to carbon dioxide extrusion (9.7 kcal mol^−1^) and indicated the potential for an equilibrium between Zn‐alkoxide and Zn‐carbonate intermediates.^[16]^ In these polymerizations, the barrier to cyclic carbonate formation is higher than that for epoxide ring‐opening, rationalizing the high polymer selectivity.^[16b]^ We hypothesized that in the absence of CO_2_, a low decarbonation barrier could increase local concentrations of metal alkoxide intermediates which might allow for epoxide formation.

To test this notion, two leading dinuclear catalysts, [LZn_2_(OAc)_2_] and [LMg_2_(OAc)_2_], were synthesized as previously reported.[^16a^, ^16c^] Poly(cyclohexene carbonate) was independently synthesized, without using carbon dioxide or the dinuclear catalyst (Figure S1).^[17]^ The PCHC used for depolymerizations shows a molar mass of 12.1 kg mol^−1^ (*Ð*=1.24) and is α,ω‐hydroxy telechelic (Figure S2).[^14^, ^18^] These features are identical to PCHC synthesized from CHO/CO_2_ ROCOP.^[16c]^ It was previously noted that CO_2_/CHO ROCOP could result in some PCHC decomposition to CHC when temperatures exceeded 80 °C, using 1 bar of CO_2_ and [LZn_2_(OAc)_2_].^[16b]^ The cyclic carbonate is proposed to form via chain back‐biting reactions from the zinc‐alkoxide intermediate (see Figure 1 for an illustration of how relative stereochemistry impacts upon backbiting). First, the extent of such backbiting during depolymerization was investigated. Thus, [LZn_2_(OAc)_2_] was reacted with 300 equivalents of PCHC (1 M solution in *p*‐xylene), at 120 °C, over 24 h, under an argon atmosphere (Table 1, Entry 1). ^1^H‐NMR spectroscopy showed that the crude product contained both the expected *trans*‐CHC (39 %, 4.0 ppm) and some cyclohexene oxide (CHO) (61 %, 3.1 ppm).

**Figure 1:**
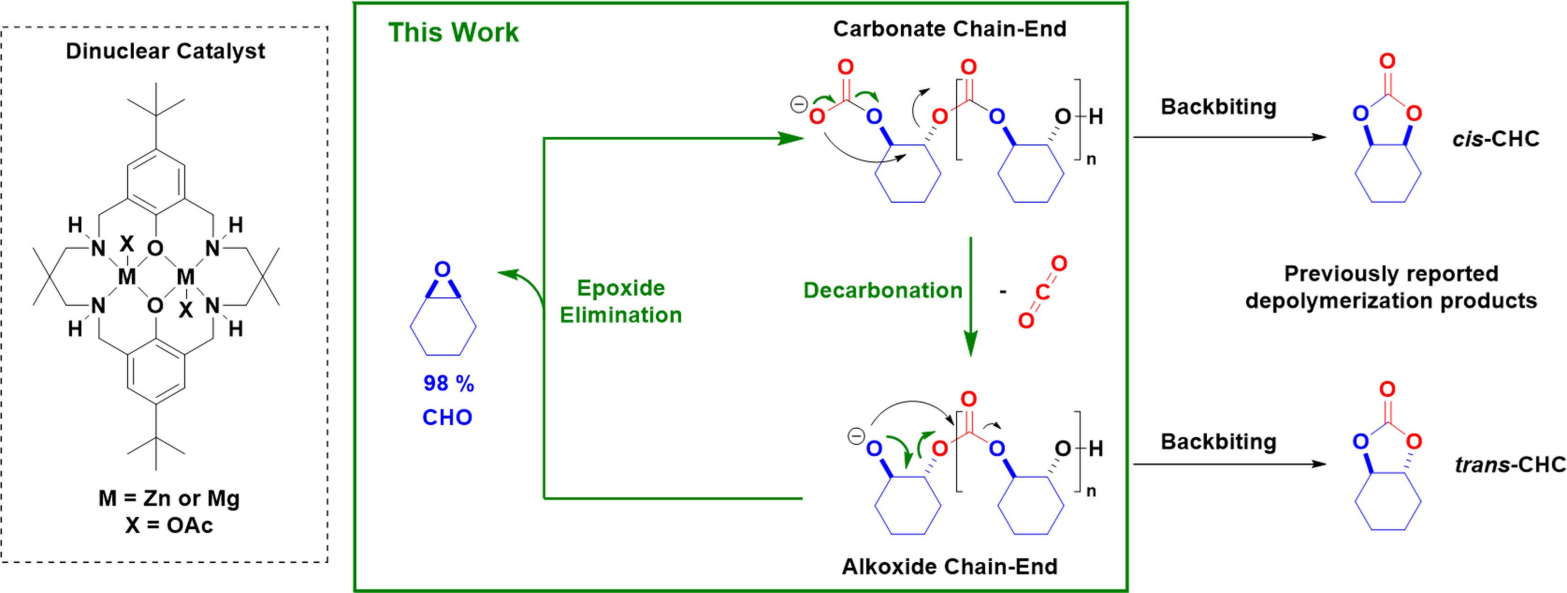
Illustration of the known depolymerizations of poly(cyclohexene carbonate), PCHC, to 5‐membered ring cyclic carbonates (*cis*‐ or *trans*‐CHC). Also illustrated is the depolymerization of PCHC to cyclohexene oxide (CHO) and carbon dioxide, reported in this work, and the structures of the dinuclear catalysts.

**Table 1:** Depolymerization of poly(cyclohexene carbonate), PCHC, using various epoxide (CHO)/CO_2_ ROCOP catalysts.^[a]^

Entry^[a]^	Catalyst	*t* [h]	PCHC Conv. [%]^[b]^	CHO [%]^[c]^	*trans*‐CHC [%]^[d]^
1	[LZn_2_(OAc)_2_]	24	94	61	39
2^[e]^	[LZn_2_(OAc)_2_]	24	90	48	52
3	[LMg_2_(OAc)_2_]	24	99	98	2
4^[f]^	[LMg_2_(OAc)_2_]	24	99	94	6
5^[g]^	[LMg_2_(OAc)_2_]	24	92	92	8
6	–	24	<5	–	–
7	[Zn(OAc)_2_]	24	47	–	>99
8	[Mg(OAc)_2_]	24	<5	–	–
9	[(salen)Cr^III^Cl]/^n^Bu_4_NN_3_	24	82	7	93
10	TBD	24	86	2	98
11	KHMDS	24	87	–	>99

[a] Reaction conditions: [PCHC]=1 M (*p*‐xylene), [cat]_0_ : [PCHC]_0_=1 : 300, 120 °C, 0.33 equiv, 1,3,5‐trimethoxybenzene (internal standard). [b] Determined from ^1^H‐NMR spectroscopy from the normalised integrals for *trans*‐CHC (4.00 ppm)+CHO (3.1 ppm) against PCHC (4.65 ppm). [c] Product selectivity determined by ^1^H‐NMR spectroscopy from the normalised integrals for CHO vs. the combined integrals for *trans*‐CHC and CHO. [d] Product selectivity determined by ^1^H‐NMR spectroscopy from the normalised integrals for *trans*‐CHC vs. the sum of the integrals for CHO and *trans*‐CHC. [e] 1 bar CO_2_. [f] PCHC synthesised from the ROCOP of CO_2_/CHO (*M*_n_=5.3 kg mol^−1^, *Ð*=1.06). [g] PCHC bought commercially from Empower Materials, QPAC130 (*M*_n_=52.4 kg mol^−1^ *Ð*=3.46). For catalyst structures see Figure S3.

To understand the product speciation, the depolymerization reaction was conducted under 1 bar CO_2_, which resulted in increased selectivity for *trans*‐CHC (52 %) (Table 1, Entry 2). As [LZn_2_(OAc)_2_] has previously been shown to be active in CO_2_/CHO ROCOP with just 1 bar of CO_2_, it is proposed that the CHO formed is then no longer innocent and can re‐enter the forward catalytic cycle. Therefore, the formation of *trans*‐CHC acts as a product ′sink′ which cannot re‐enter the cycle (Figure 1).

To understand the influence of the catalyst, the reaction was repeated under otherwise identical conditions but using [LMg_2_(OAc)_2_]. Using the di‐Mg^II^ catalyst significantly increased the depolymerization selectivity for cyclohexene oxide (98 %) (Table 1 Entry 3). The same di‐Mg^II^ catalyst operates in forward polymerization at higher temperatures, with minimal *trans*‐CHC observed even at 140 °C, which indicates it has a higher barrier to cyclic carbonate formation compared to the di‐zinc analogue.^[16c]^ The di‐Mg^II^ catalyst was also used to depolymerize PCHC made from the ROCOP of CO_2_/CHO using the same di‐Mg^II^ catalyst (*M*_n_=5.3 kg mol^−1^, *Ð*=1.06) and a commercial sample from Empower Materials, QPAC130. The di‐Mg^II^ catalyst performed comparably in the depolymerization of these materials and of the PCHC synthesized from ROP catalysis (Table 1 Entry 4 and 5). No depolymerization occurred in the absence of catalyst (Table 1, Entry 6). Reactions using Mg(OAc)_2_ or Zn(OAc)_2_ as catalysts resulted only in *trans*‐CHC without any CHO formation (Table 1, Entries 7 and 8). Previously, Darensbourg and team reported the thermal decomposition of PCHC to *trans*‐CHC using a [(salen)Cr^III^Cl]/PPNN_3_ catalyst system.^[10a]^ Using our depolymerization conditions, the catalyst system behaved equivalently and formed only *trans*‐CHC (93 %, Table 1, Entry 9). Given the success of TBD and bis(trimethylsilyl)amide (HMDS) in the depolymerization of poly(limonene carbonate)^[10b]^ and poly(cyclopentene carbonate),^[10a]^ both were tested using PCHC. Both catalysts formed only *trans*‐CHC which is consistent with the bases deprotonating the hydroxy‐polymer chain end groups to form alkoxide moieties (Table 1, Entry 10 and 11). Darensbourg and co‐workers have previously proposed that such “free” alkoxide groups favor cyclic carbonate formation.^[9]^ These results using other catalysts demonstrate the importance of the dinuclear Zn^II^ or Mg^II^ catalysts for selective PCHC depolymerization to CHO.

To better understand the high selectivity of the di‐Mg^II^ catalyst, PCHC depolymerization was monitored using ^1^H‐NMR spectroscopy (using an internal standard). This technique allowed for comparisons of the relative conversions of PCHC, CHO and CHC, respectively (Figure 2a, Table S1). The PCHC concentration decreased exponentially reaching 87 % conversion over 8 h and, at the same time, the CHO conversion increased exponentially. The observed rate constant (*k*_obs_) for PCHC consumption and CHO formation were similar at 0.407 h^−1^ and 0.361 h^−1^, respectively (Figure S7). The data also indicated a brief initiation period, ≈20 mins, prior to any conversion. It is tentatively proposed that during this time the polymer chain hydroxy groups react with the catalyst to form an active metal‐alkoxide initiator for depolymerization. The di‐Mg^II^ catalyst shows good depolymerization activity, reaching a turn‐over‐frequency of 150 h^−1^ in the first 40 mins of reaction. The selectivity for cyclohexene oxide remains very high throughout the reaction, with low quantities of *trans*‐CHC being observed (6 %). The depolymerization was repeated with regular removal of aliquots which were analyzed using size‐exclusion chromatography (SEC) to monitor the PCHC molar mass evolution (Figure 2b). The polymer showed a steady decrease in molar mass with concomitant increase in dispersity, until around 6 kg mol^−1^ after which it did not appear to decrease further. Since no plateau in conversion vs. time data is observed using NMR spectroscopy (Table S1), it is proposed that SEC is unable to discriminate the lower molar mass fractions which all elute at the same time.

**Figure 2:**
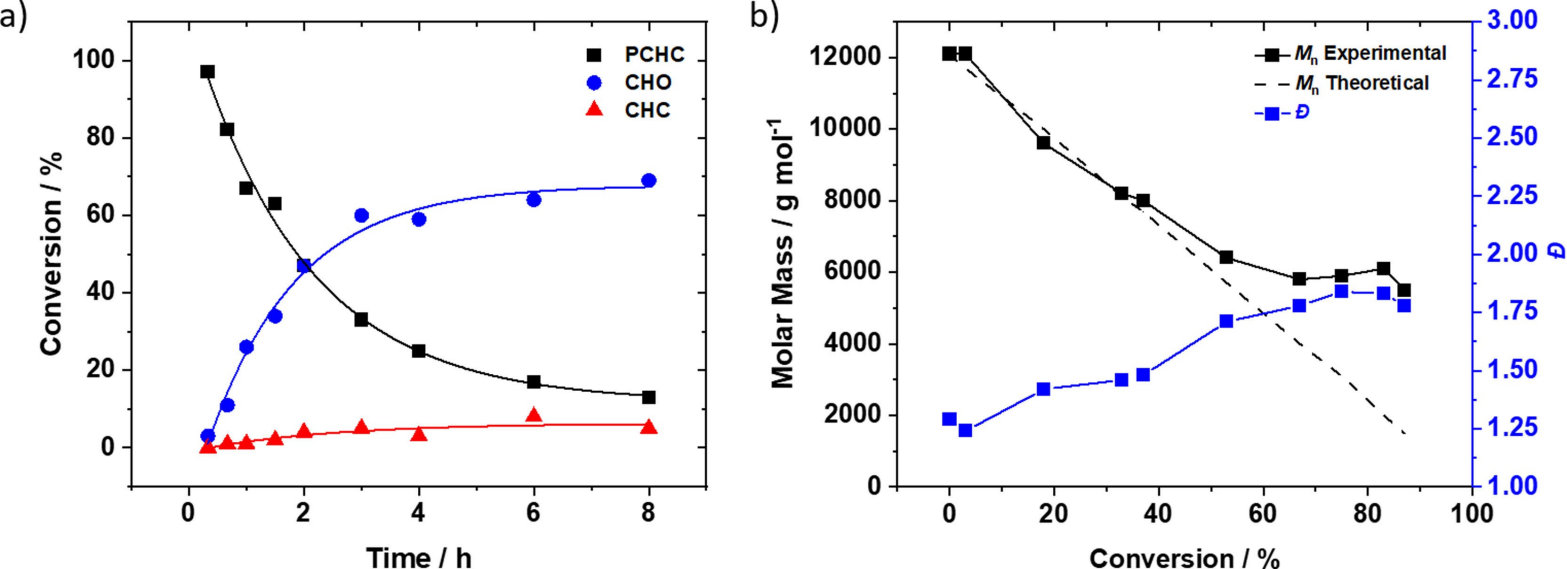
Depolymerization reaction data, under standard conditions (0.3 mol % catalyst, 1 M PCHC in *p*‐xylene, 120 °C, Ar). a) Conversion vs. time data for PCHC (black squares), CHO (blue circles) and *trans*‐CHC (red triangles). b) Evolution of PCHC molar mass (*M*_n, SEC_ black squares) and dispersity (*Ð*, blue squares) vs. depolymerization reaction conversion.

The depolymerization mechanism could occur either directly from PCHC or by a catalyzed decomposition of *trans*‐CHC. To distinguish between these routes, a sample of pure *trans*‐CHC was synthesized by cycloaddition of *trans*‐1,2‐cyclohexane diol with carbon dioxide, using 2,2,6,6‐tetramethyl pyridine and tosyl chloride (Figure S8 and S9).^[19]^ The *trans*‐CHC was reacted with [LMg_2_(OAc)_2_], under the same conditions as used in depolymerization reactions (0.3 mol %, 120 °C, p‐xylene). The reaction did not result in any significant conversion and only trace CHO was detected after 24 h (<5 %). During the PCHC depolymerization reactions, the overall concentration of *trans*‐CHC is around 100 times lower than in the control experiment. This indicates that the formation of CHO through decarbonation of *trans*‐CHC is very unlikely to occur in depolymerizations. Rather, CHO is proposed to form by PCHC depolymerization with only low quantities of *trans*‐CHC forming by alkoxide back‐biting reactions.

To understand whether the depolymerization occurred by random or chain end mechanisms, an end‐capped polymer sample was tested. Thus, α,ω‐hydroxy telechelic PCHC (PCHC‐OH) was end‐capped with trifluoroacetyl groups (PCHC‐O_2_CCF_3_) by reaction with excess trifluoroacetic anhydride (Figure S10 and S11). After this end‐capping reaction, ^31^P{^1^H} NMR spectroscopy was used to titrate residual hydroxy end‐groups — the technique indicated >95 % conversion to end‐capped chains (Figure S12). Subjecting the end‐capped polymer to the same depolymerization conditions failed to yield any epoxide or cyclic carbonate (Table S2). This observation supports a chain‐end catalyzed depolymerization mechanism.

A theoretical investigation using DFT allowed for comparison of the different pathways (Figure 3 and S13). The di‐Mg^II^ alkoxide intermediate (**INT1**) was set as the reference structure (Δ*G*=0.0 kcal mol^−1^) for these calculations. It can undergo an S_N_2 attack on an adjacent polymer chain methine group resulting in the elimination of CHO and formation of a di‐Mg^II^ carbonate intermediate. The epoxide formation shows a calculated transition state barrier of 19.2 kcal mol^−1^. The di‐Mg^II^ alkoxide intermediate (**INT1**) can be reformed through decarbonation which has a significantly lower barrier (Figure 1, ≈9 kcal mol^−1^). Alternatively, the di‐Mg^II^ alkoxide intermediate (**INT1**) can pre‐organize itself, via a ring‐flip of the cyclohexyl ring (**INT3**), to position itself for nucleophilic attack upon an adjacent polymer chain carbonyl group. A 2‐step addition‐elimination reaction (**TS2**) generates the *trans*‐cyclohexene carbonate (**INT4**). This backbiting process occurs with a higher transition state barrier of 25.5 kcal mol^−1^. The difference between the transition state barriers for the two products formed from **INT1** is 6.3 kcal mol^−1^, which is consistent with the high experimental selectivity for cyclohexene oxide formation (Figures 3 and S13).

**Figure 3:**
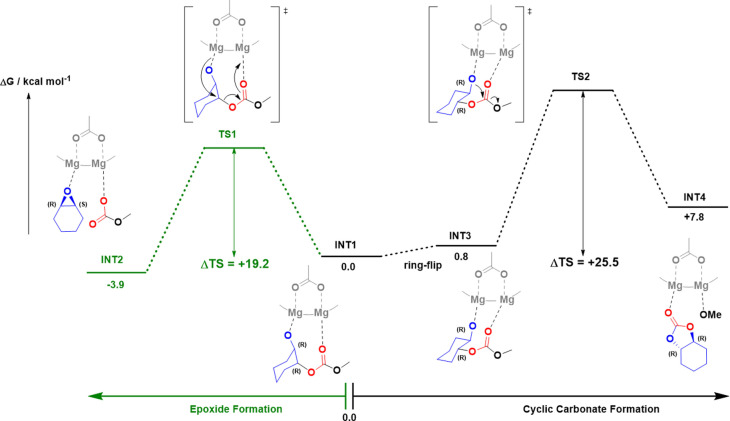
Illustration of the potential energy surface for the ring‐closing depolymerization of PCHC forming both CHO and *trans*‐CHC using a di‐Mg^II^ catalyst (see Figure S13 for the full DFT investigation).

In conclusion, a di‐Mg^II^ catalyst shows efficient poly(cyclohexene carbonate) depolymerization to cyclohexene oxide for the first time. The reaction occurs with high selectivity (>98 %) and activity (TOF=150 h^−1^). Successful depolymerization to epoxide is dependent upon catalyst selection and is not observed using control complexes or other (de)polymerization catalysts. The new method of PCHC chemical recycling is expected to benefit its future application as an engineering plastic and may help to reduce the embedded energy associated with virgin epoxides. In future, this work should inspire investigation of other di‐ and multinuclear catalysts to understand the depolymerization mechanism.

## Conflict of interest

CKW is a director of Econic Technologies.

## Supporting information

As a service to our authors and readers, this journal provides supporting information supplied by the authors. Such materials are peer reviewed and may be re‐organized for online delivery, but are not copy‐edited or typeset. Technical support issues arising from supporting information (other than missing files) should be addressed to the authors.

Supporting Information

## Data Availability

The data that support the findings of this study are available in the Supporting Information of this article.
