# Supplementary material for: Chemical Recycling of Poly(Cyclohexene Carbonate) Using a Di‐MgII Catalyst
Source: Angew Chem Int Ed Engl. 2022 May 5;61(26):e202201785. doi: 10.1002/anie.202201785 (PMC9322669; doi:10.1002/anie.202201785)
Supplement: Supplementary file 1 — Supporting Information [file ANIE-61-0-s001.pdf]

Angewandte Chemie
Eine Zeitschrift der Gesellschaft Deutscher Chemiker

Supporting Information

Chemical Recycling of Poly(Cyclohexene Carbonate) Using a Di-Mg^{II} Catalyst

F. N. Singer, A. C. Deacy, T. M. McGuire, C. K. Williams, A. Buchard**

Author Contributions

F.S. Conceptualization:Supporting; Formal analysis:Equal; Investigation:Lead; Methodology:Equal; Writing – original draft:Equal
A.D. Formal analysis:Equal; Investigation:Supporting; Methodology:Equal; Supervision:Supporting; Writing – original draft:Supporting; Writing – review & editing:Lead
T.M. Conceptualization:Supporting; Data curation:Supporting; Formal analysis:Supporting; Investigation:Supporting; Methodology:Supporting; Software:Supporting; Writing – original draft:Supporting; Writing – review & editing:Supporting
C.W. Conceptualization:Lead; Data curation:Equal; Formal analysis:Supporting; Funding acquisition:Lead; Investigation:Supporting; Methodology:Supporting; Project administration:Equal; Resources:Equal; Supervision:Lead; Validation:Lead; Writing – original draft:Supporting; Writing – review & editing:Lead
A.B. Conceptualization:Lead; Data curation:Equal; Formal analysis:Supporting; Funding acquisition:Lead; Investigation:Supporting; Methodology:Supporting; Project administration:Equal; Resources:Equal; Software:Supporting; Supervision:Lead; Validation:Lead; Writing – original draft:Supporting; Writing – review & editing:Lead

Supporting Information

Table of Contents

Synthetic Procedures S3

Trans-1,2-cyclohexene carbonate (*trans*-CHC) S3

Poly(cyclohexene carbonate) (PCHC-OH) S3

Trifluoroacetate end-capped poly(cyclohexene carbonate) (PCHC-O_2CCF_3) S4

General depolymerization experimental procedure S4

^{31}P NMR spectroscopy end-group titration S4

Additional Information S5

Scheme S1: Illustration of the chemical recycling of CO_2-polycarbonates to epoxides reported in the literature. S5

Figure S1: ^{1}H NMR spectrum of PCHC-OH ($CDCl_3$, 298 K). S6

Figure S2: ^{31}P{^{1}H} NMR spectrum ($CDCl_3$, 298 K) used for end-group titration of PCHC S7

Figure S3: Illustration of literature catalysts used in depolymerization of PCHC to CHO. S8

Figure S4: Representative ^{1}H NMR spectrum ($CDCl_3$, 298 K) of the depolymerization of PCHC S9

Figure S5: Representative ^{1}H NMR spectrum (d8-toluene, 298 K) of the depolymerization of PCHC at low conversion S10

Figure S6: Representative ^{1}H NMR spectrum (d8-toluene, 298 K) of the depolymerization of PCHC at high conversion. S10

Table S1: Experimental data for the depolymerization of PCHC using [$LMg_2(OAc)_2$] shown in Figure 2 of the main article. S11

Figure S7: Semi-log plot of time vs $\ln([PCHC]_0/[PCHC]_t)$ and vs $\ln([PCHC]_0/([PCHC]_0[CHO]_t))$ S12

Figure S8: ^{1}H NMR spectrum trans-CHC ($CDCl_3$, 298 K). S13

Figure S9: ^{13}C{^{1}H} NMR spectrum of trans-CHC ($CDCl_3$, 298 K) S13

Figure S10: ^{1}H NMR spectrum of PCHC-O_2CCF_3 ($CDCl_3$, 298 K). S14

Figure S11: ^{19}F{^{1}H} NMR spectrum of PCHC-O_2CCF_3 ($CDCl_3$, 298 K) S15

Figure S12: ^{31}P{^{1}H} NMR spectrum used for end-group titration of PCHC-O_2CCF_3 ($CDCl_3$, 298 K). S16

Table S2: Depolymerization of PCHC-O_2CCF_3 using $LMg_2(OAc)_2$. S17

Computational Modelling S18

Density Functional Theory calculations details S18

Figure S13: Computed free-enthalpy profile for the ring closing of PCHC to CHO and the backbiting to *trans*-CHC. S19

Table S3: Computed Free Gibbs Energy of structures given in Fig. S13. .... S20

Energy span calculations S20

References S21

General Experimental Details

Where stated, manipulations were performed under an atmosphere of argon, using standard Schlenk or glovebox techniques. All catalysts and reagents were purchased from Merck, unless stated otherwise. 2,2,6,6-Tetramethylpiperidine was used as received. 1,5,7-Triazabicyclo[4.4.0]dec-5-ene was dissolved in anhydrous THF and filtered through celite, followed by recrystallization, from hot toluene, and dried under vacuum. Trans-1,2-cyclohexane diol was crystallized from ethyl acetate and dried under vacuum. 1,3,5-Trimethoxybenzene was crystallized from hexane and dried *in vacuo*. Anhydrous p-xylene was degassed, by bubbling argon through the solution for at least 1 h, and stored over 4 Å molecular sieves, under argon. Both [$LZn_2(OAc)_2$] and [$LMg_2(OAc)_2$] were synthesized using literature procedures.[1]

NMR spectra were recorded on a Bruker 400 or 500 MHz spectrometer and were referenced relative to the residual protonated solvent signal. Size exclusion chromatography (SEC) was performed using THF as eluent, with polymer samples of a concentration 1–4 mg mL^{-1}. The samples were run on an Agilent 1260 Infinity series instrument, at 1 mL min^{-1}, using two PLgel 5 µm MIXED-D 300 x 7.5 mm columns in series. Samples were detected with a differential refractive index (RI) detector. Calibration was performed with 11 narrow molecular weight polystyrene standards (M_w 0.62-260.9 kg mol^{-1}).

Synthetic Procedures

Trans-1,2-cyclohexene carbonate (*trans*-CHC).[2]

Trans-1,2-cyclohexane diol (5.00 g, 43.1 mmol), 4-toluenesulfonyl chloride (8.20 g, 43.0 mmol) and anhydrous MeCN (140 mL) were combined in a Schlenk flask under argon. The atmosphere was exchanged for CO_2 and the mixture cooled to 0 °C. 2,2,6,6-Tetramethylpiperidine (14.7 mL, 86.1 mmol) was added dropwise, over 5 minutes. The reaction was held at 0 °C for a further 15 minutes and then allowed to warm to room temperature and stirred for 20 hours. A white precipitate formed and was removed by filtration and washed with MeCN (3 x 80 mL). The filtrate was reduced *in vacuo* and the residue dissolved in a mixture of hexane and ethyl acetate (1:1, 100 mL) and filtered. The filtrate was reduced *in vacuo* and purified by gradient hexane/ethyl acetate column chromatography (10/1 to 4/1). The product fractions were combined and reduced *in vacuo*. The residue was dissolved in toluene (40 mL) and hexane (350 mL). The solution was cooled to -20 °C to induce crystallization. The white needles were isolated by filtration and dried *in vacuo*, at 40 °C for 3 hours. (3.50 g, 24.6 mmol, 57 %) ^{1}H NMR (400 MHz, CDCl3, δ in ppm) 4.08 – 3.94 (m, 2H) 2.33 – 2.19 (m 2H) 2.01 – 1.83 (m, 2H) 1.76 – 1.59 (m, 2H) 1.50 – 1.32 (m, 2H) ^{13}C{^{1}H} NMR (101 MHz, CDCl3, δ in ppm) 155.1, 83.6, 28.3, 23.2.

Poly(cyclohexene carbonate) (PCHC-OH)

Trans-cyclohexene carbonate (2.99 g, 21.0 mmol) and trans-1,2-cyclohexanediol (0.03 g, 21 µmol) were combined in an ampoule with anhydrous toluene (5 mL). A solution of 1,5,7-triazabicyclo[4.4.0]dec-5-ene in toluene (0.25 mL, 0.168 M, 42 µmol) was added *via* syringe. The ampoule was sealed under argon and heated to 60 °C, for 8 hours. The reaction was quenched by adding DCM (1 mL) and exposure to air. The solvent was removed *in vacuo* and the residue dissolved in DCM (20 mL). The solution was added dropwise through a syringe filter to a stirred solution of 1 M HCl, in MeOH (250 mL). The solid was isolated either via filtration or centrifugation and the process repeated twice more. The resulting white solid was dried under vacuum at 60 °C for 4 hours. (2.02 g,

12.8 mmol, 68 %) ^{1}H NMR (400 MHz, $CDCl_3$, δ in ppm) 4.75–4.51 (br, 2 H), 2.21–1.97 (br, 2H), 1.80 – 1.61 (br, 2H), 1.61 – 1.12 (br, 4H).

Trifluoroacetate end-capped poly(cyclohexene carbonate) (PCHC-O_2CCF_3)

During the synthesis of PCHC-OH, after heating at 60 °C for 8 hours trifluoroacetic anhydride (20 wt % vs cyclic carbonate) was added under argon and allowed to react for 1 hour. Polymer work-up followed the same procedure for PCHC-OH. (102 mg, 0.82 mmol, 41 %) ^{1}H NMR (400 MHz, $CDCl_3$, δ in ppm) 4.73–4.48 (br, 2 H), 2.23 – 1.96 (br, 2H), 1.81 – 1.62 (br, 2H), 1.56 – 1.14 (br, 4H). ^{19}F{^{1}H} NMR (471 MHz, $CDCl_3$, δ in ppm) −75.10 (s), −75.23 (s).

General depolymerization experimental procedure

PCHC (0.5-1.0 mmol) and 1,3,5-trimethoxybenzene (0.33 equiv.) were combined in a flask with p-xylene, in a glovebox, under an argon atmosphere. The catalyst was added and the reaction mixture heated in a PTFE capped vial, under argon to 120 °C. Reactions were quenched by cooling in ice and exposing to air.

^{31}P NMR spectroscopy end-group titration

Following an adapted literature procedure:[3] in the glovebox, a 10.0 mL stock solution of bisphenol A (400 mg) and chromium triacetate (6 mg) in anhydrous pyridine was prepared. PCHC (40 mg) was dissolved in deuterated chloroform and the bisphenol A stock solution (40 µL) was added. 2-Chloro-4,4,5,5-tetramethyl dioxaphospholane (excess, 40 µL) was charged to the reaction vessel and the reaction solution was left overnight. The reaction solution was transferred to a J-Youngs NMR tube for data collection. *N.B. To ensure the data was quantitative, ^{31}P NMR spectroscopy was recorded with a 10s T1 delay.*

Additional Information

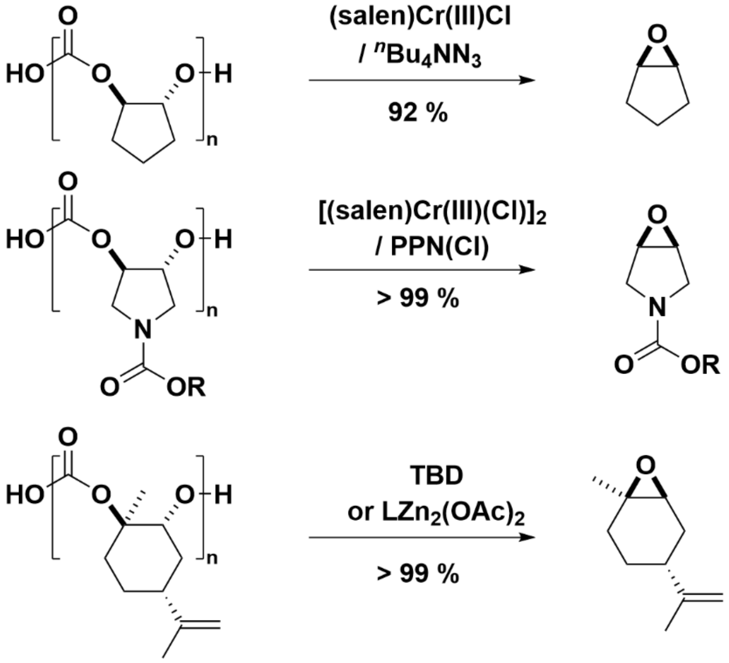

Scheme S1: Illustration of the chemical recycling of CO_2-polycarbonates to epoxides reported in the literature.

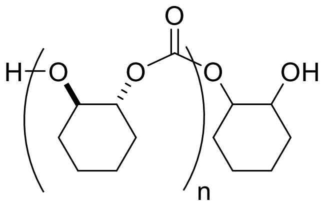

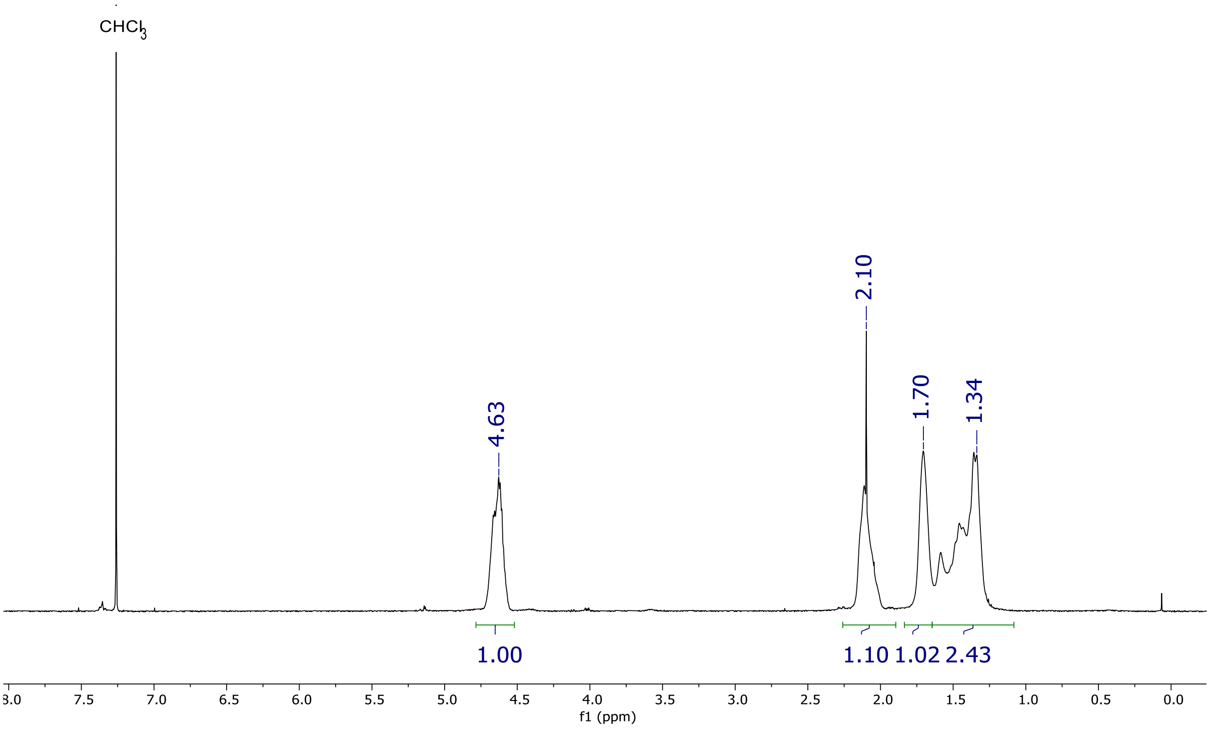

Figure S1: ^{1}H NMR spectrum of PCHC-OH ($CDCl_3$, 298 K).

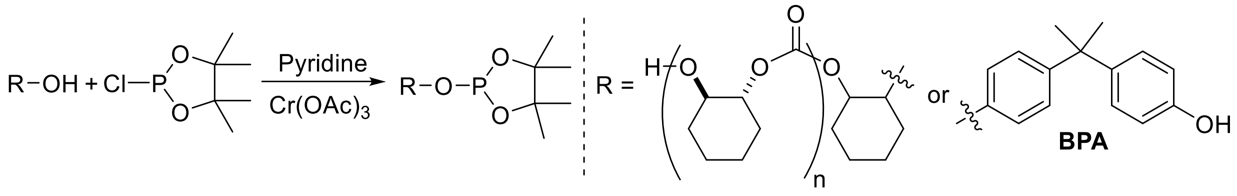

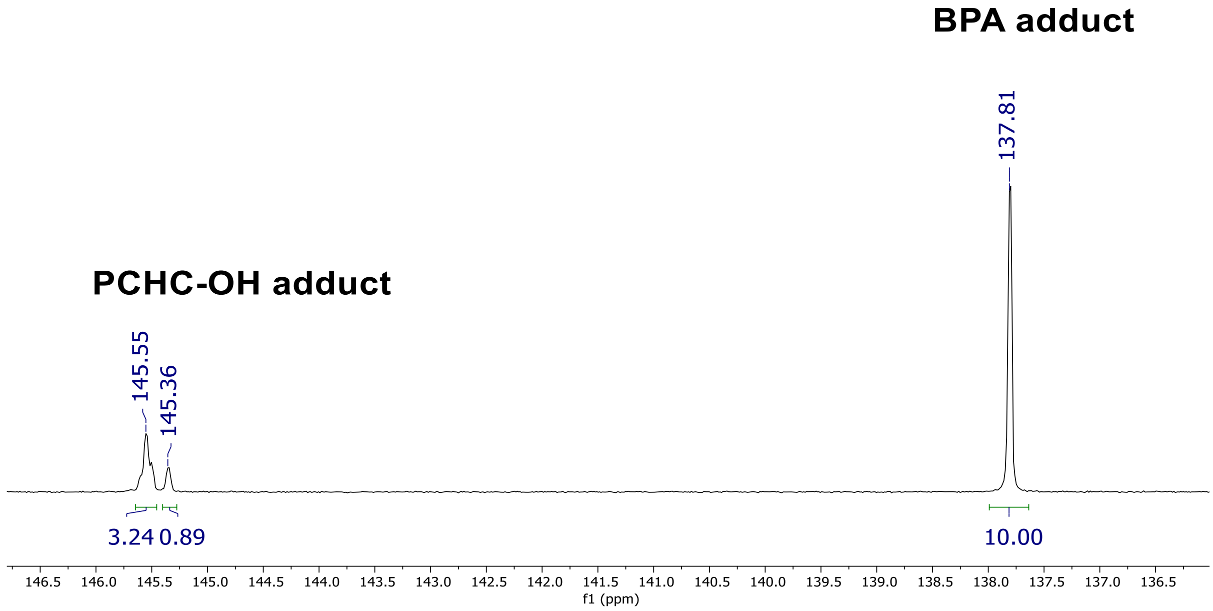

Figure S2: $^{31}P\{^{1}H\}$ NMR spectrum ($CDCl_3$, 298 K) used for end-group titration of PCHC. Resonances at 145.3-145.5 are derived from the reaction of 2-chloro-4,4,5,5-tetramethyl dioxaphospholane with terminal-OH groups on PCHC and has been reported previously.[3]

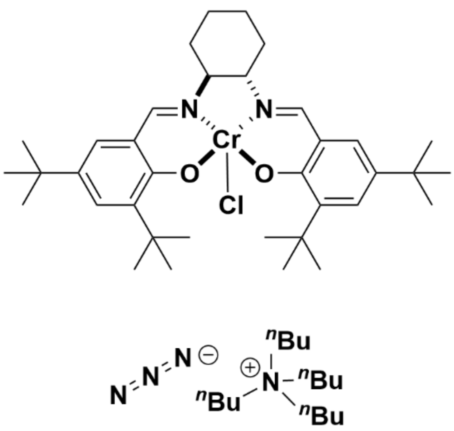

(salen)Cr(III)Cl / $^{n}Bu_4NN_3$

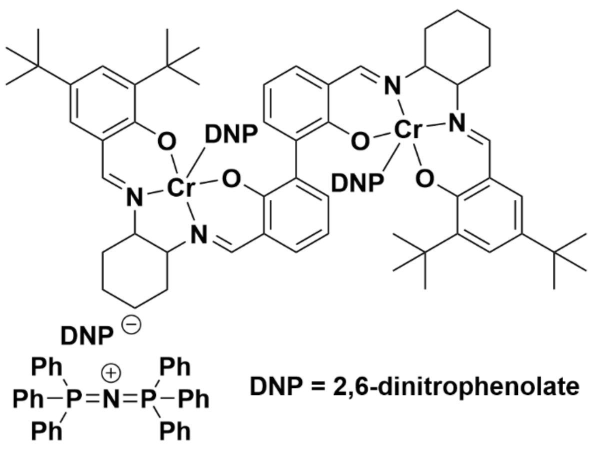

[(salen)Cr(III)DNP]$_2$ / PPN(DNP)

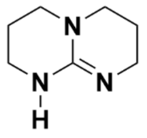

TBD = triazabicyclo[4.4.0]dec-5-ene

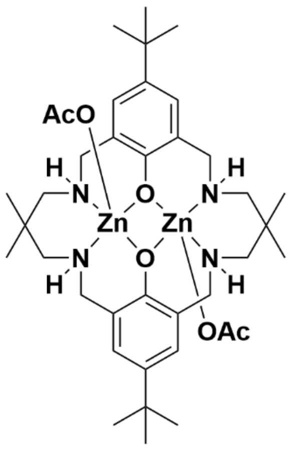

$LZn_2(OAc)_2$

Figure S3: Illustration of literature catalysts used in depolymerization of PCHC to CHO.

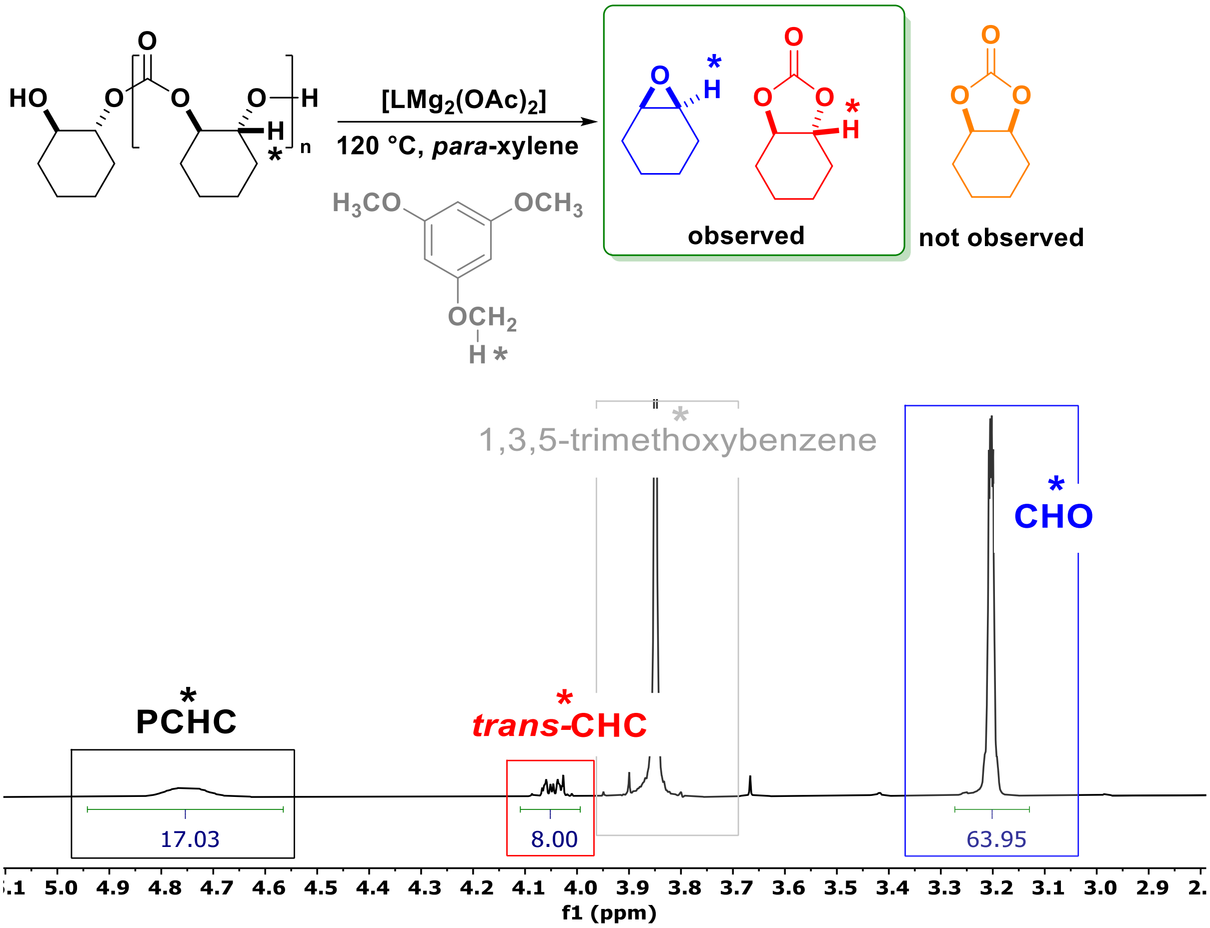

Figure S4: Representative ^{1}H NMR spectrum ($CDCl_3$, 298 K) of the depolymerization of PCHC showing asterisked protons (Table S1, entry 9).

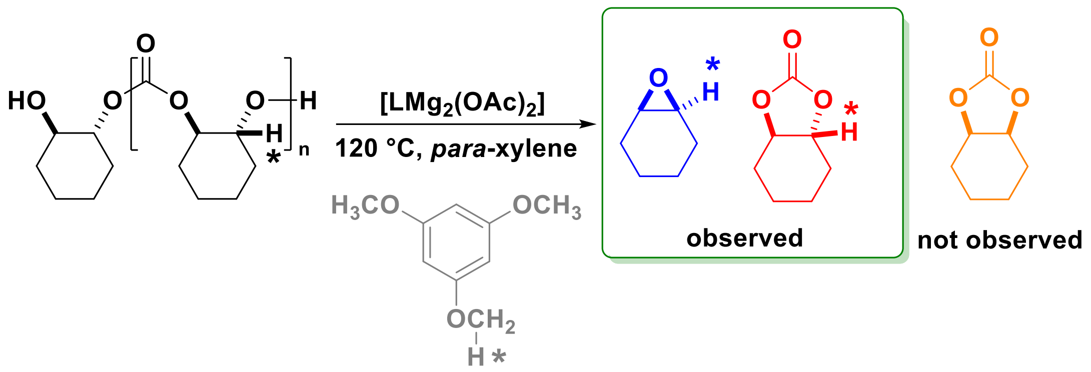

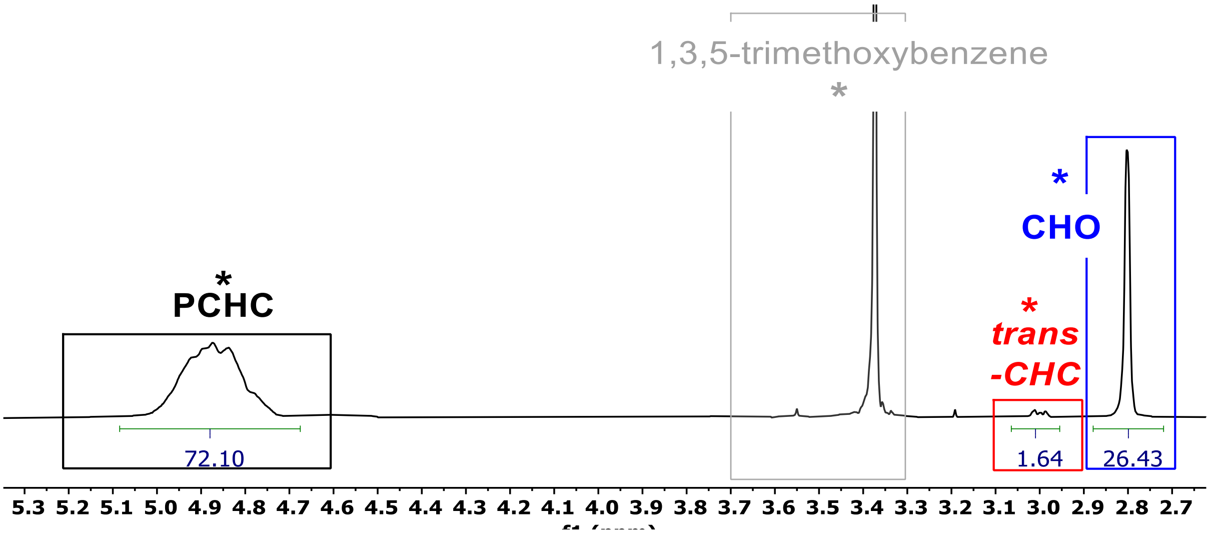

Figure S5: Representative ^{1}H NMR spectrum (d8-toluene, 298 K) of the depolymerization of PCHC at low conversion showing asterisked protons. N.B. no cis-CHC observed (expected resonance δ = 3.75 ppm).

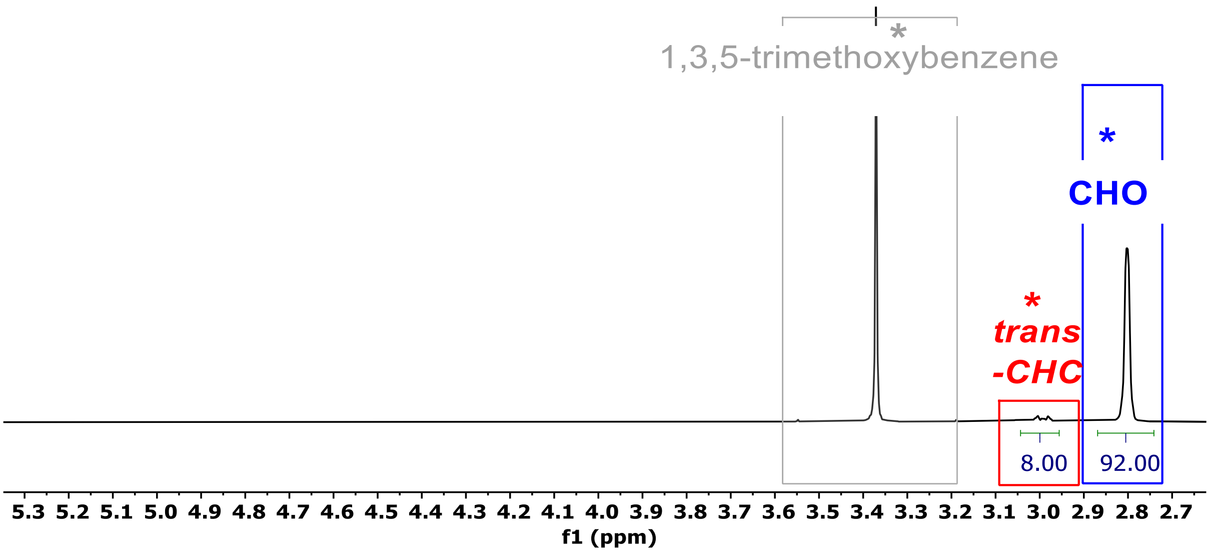

Figure S6: Representative ^{1}H NMR spectrum (d8-toluene, 298 K) of the depolymerization of PCHC at high conversion showing asterisked protons. asterisked protons. N.B. no cis-CHC observed (expected resonance δ = 3.75 ppm).

Table S1: Experimental data for the depolymerization of PCHC using [$LMg_2(OAc)_2$] shown in Figure 2 of the main article.[a]

Entry	Time / h	Conv. of PCHC / %[b]	CHO / %[b]	CHC / %[b]	TOF / h^{-1}[c]	M_n NMR / g mol^{-1}[d]	M_n SEC / g mol^{-1}[e]	Đ[f]
1	0	0	0	0	-	8400	12100	1.29
2	0.33	3	3	0	-	8200	12100	1.24
3	0.67	18	11	1	100	6900	9600	1.42
4	1	33	26	1	149	5600	8200	1.46
5	1.5	37	34	2	95	5300	8000	1.48
6	2	53	47	4	95	3900	6400	1.71
7	3	67	60	5	75	2800	5800	1.78
8	4	75	59	5	61	2100	5900	1.84
9	6	83	64	8	44	1400	6100	1.83
10	8	87	69	8	34	1100	5500	1.78

[a]Reaction conditions: $[PCHC]_0$: $[Cat]_0$ = 300 : 1, $[PCHC]_0$ = 1 M in *p*-xylene ,120 °C.[b] Determined by ^1H NMR spectroscopy ($CDCl_3$) using the normalised integration of PCHC (δ = 4.65 ppm), *trans*-CHC (δ = 4.00 ppm) and CHO (δ = 3.12 ppm), against an internal standard of 1,3,5-trimethoxybenzene (δ = 6.01 ppm).[c] TOF = moles of CHO formed/time*moles of catalyst (initiation time of 20mins have been removed).[d] Determined by ^1H NMR spectroscopy ($CDCl_3$) using the relative integration of PCHC and end groups (δ = 3.65 ppm).[e] Calculated by SEC relative to polystyrene standards in tetrahydrofuran (THF) eluent.[f] Đ=M_w/M_n.

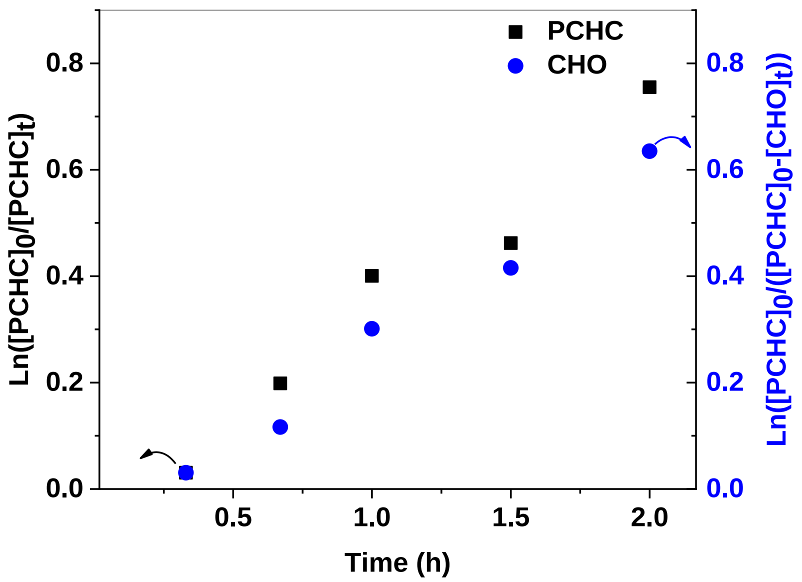

Figure S7: Semi-log plot of time *vs* ln($[PCHC]_0/[PCHC]_t$) (black, left y-axis) and *vs* ln($[PCHC]_0/([PCHC]_0[CHO]_t)$) (blue, right y-axis) for 0-50% conversion of PCHC. The reaction was conducted at $[PCHC]_0$: $[[LMg_2(OAc)_2]]_0$ = 300 : 1, $[PCHC]_0$ = 1 M in p-xylene ,120 °C (Table S1). k_{obs} values were determined from the linear fits to both PCHC and CHO data with $[PCHC]_t$ and $[CHO]_t$ determined by normalised ^{1}H NMR spectroscopy using the integration of PCHC resonances of PCHC and CHO resonances, respectively. For PCHC depolymerization, k_{obs} = 0.407 h^{-1}. For CHO formation, the k_{obs} = 0.361 h^{-1}. The slight difference in the two rates is accounted for by the concomitant formation of *trans*-CHC.

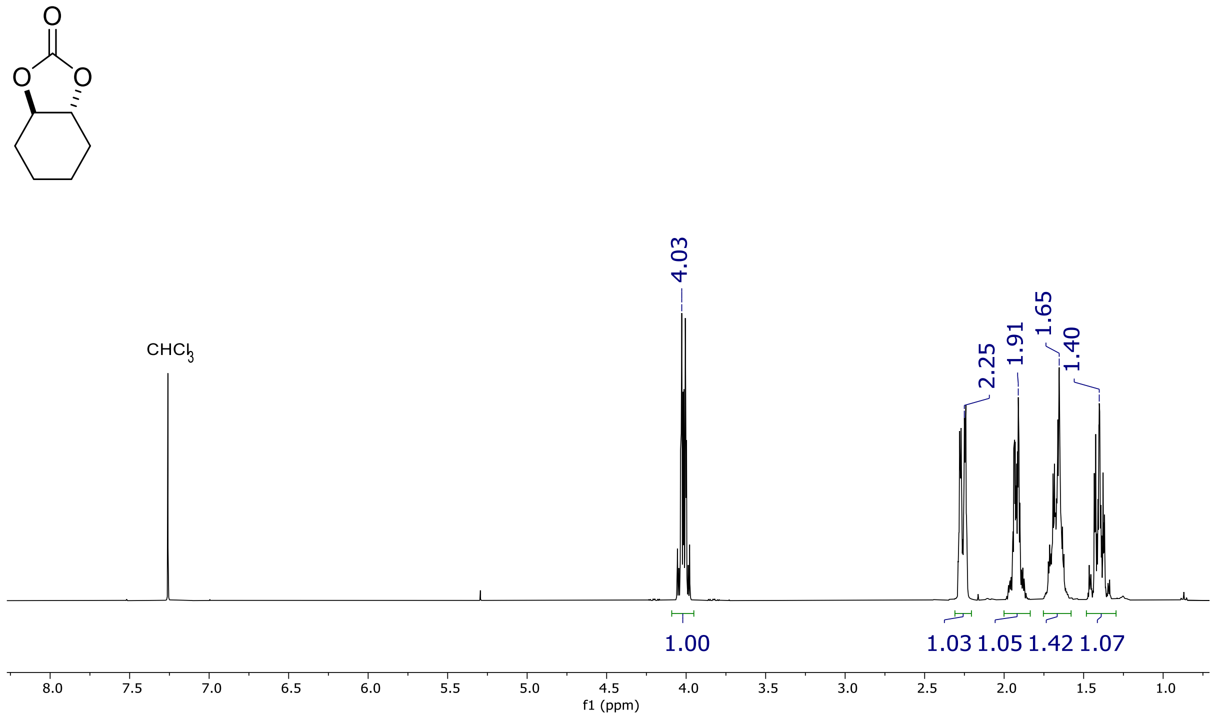

Figure S8: ^{1}H NMR spectrum *trans*-CHC ($CDCl_3$, 298 K).

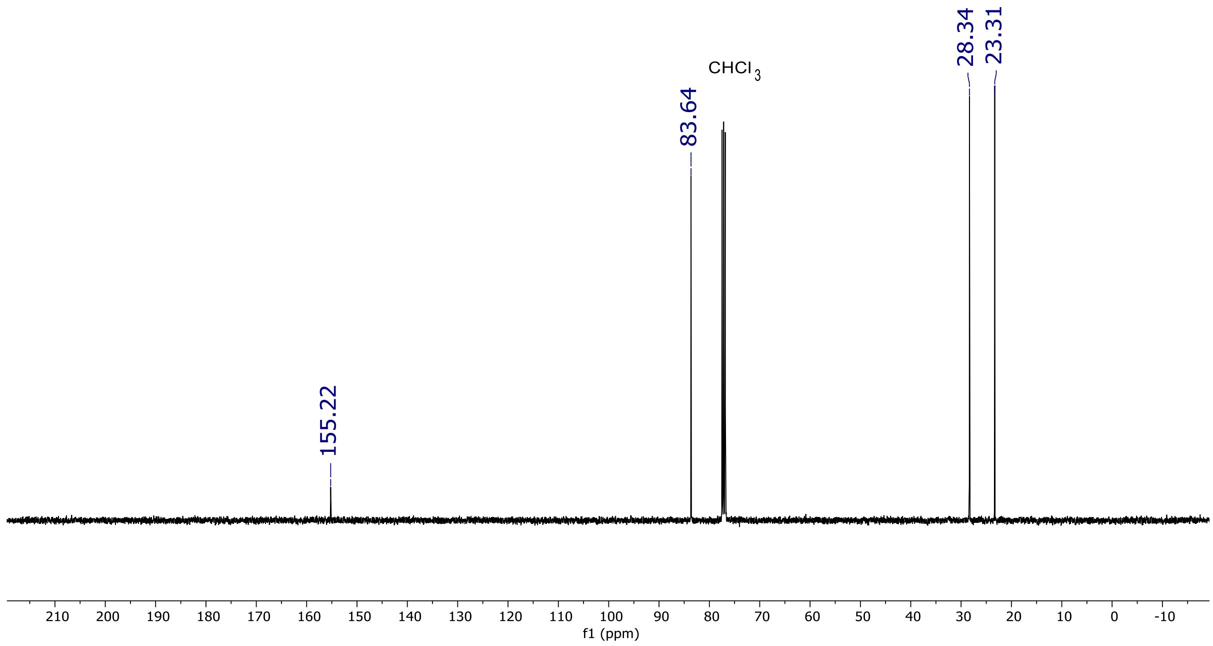

Figure S9: ^{13}C{^{1}H} NMR spectrum of *trans*-CHC ($CDCl_3$, 298 K).

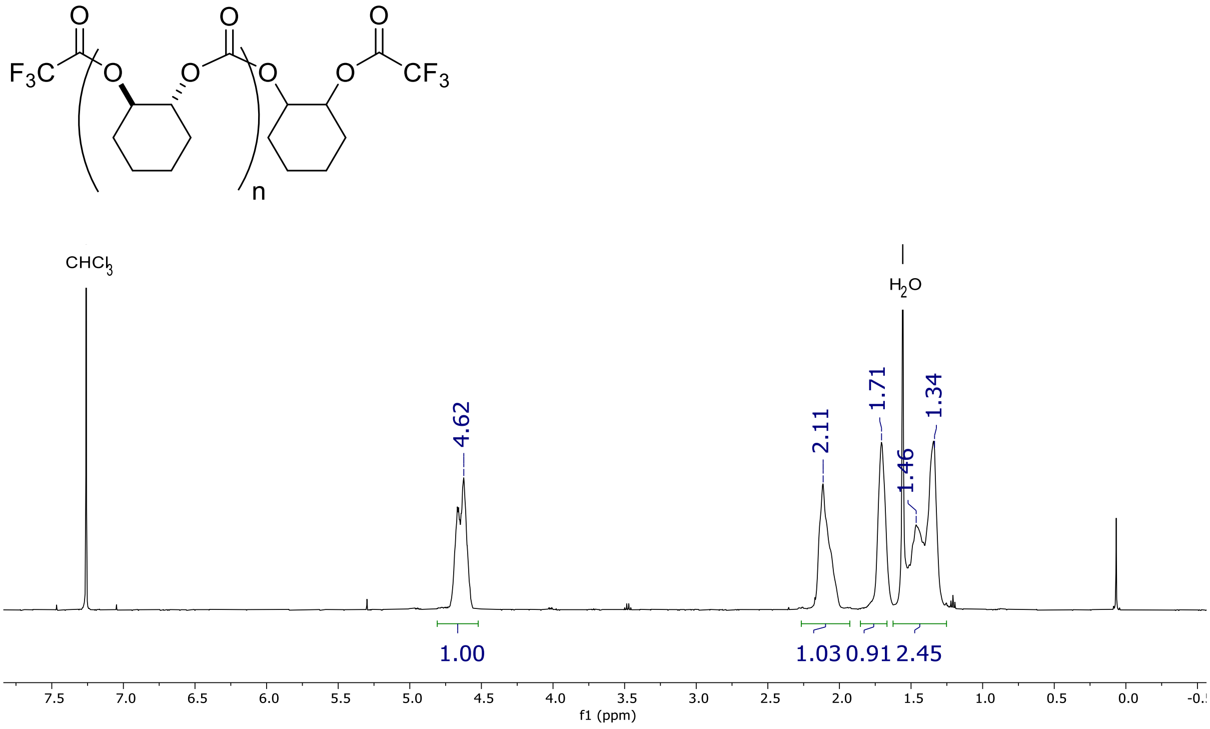

Figure S10: ^{1}H NMR spectrum of PCHC-O_2CCF_3 ($CDCl_3$, 298 K). PCHC prepared from ROP of *trans*-CHC followed by end-capping using trifluoroacetic anhydride.

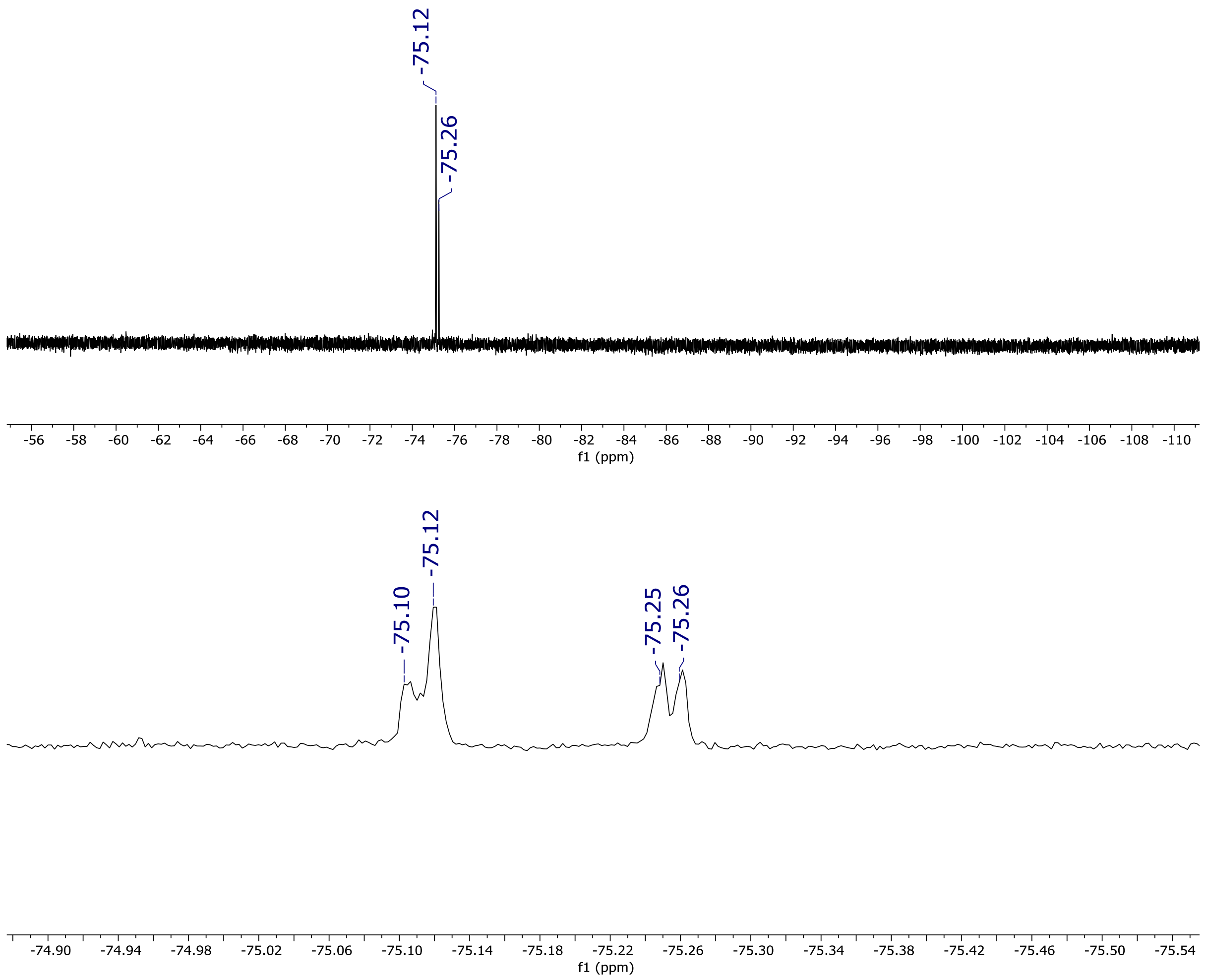

Figure S11: $^{19}F\{^{1}H\}$ NMR spectrum of PCHC-O_2CCF_3 ($CDCl_3$, 298 K). PCHC prepared from ROP of *trans*-CHC followed by end-capping using trifluoroacetic anhydride.

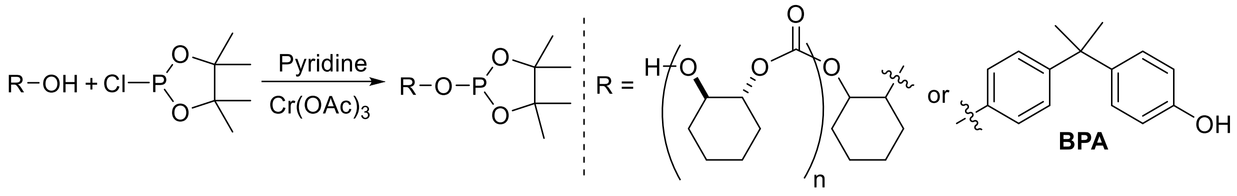

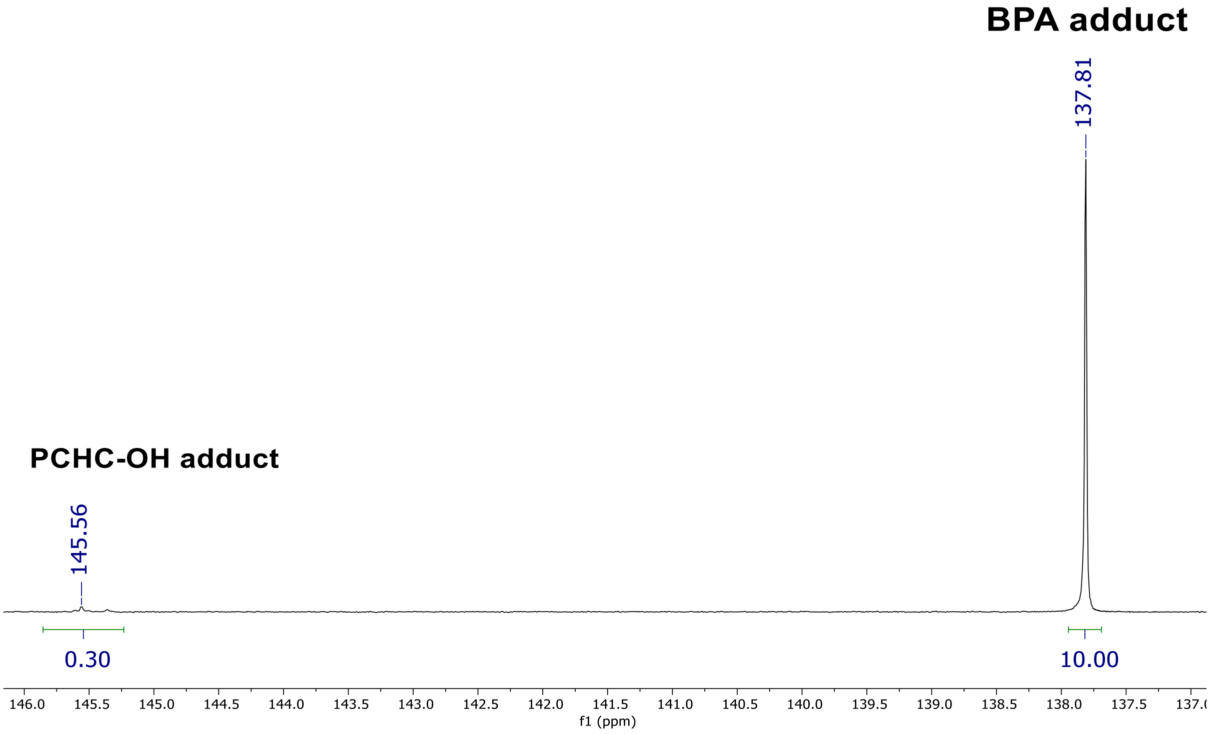

Figure S12: $^{31}P\{^{1}H\}$ NMR spectrum used for end-group titration of PCHC-O_2CCF_3 ($CDCl_3$, 298 K). Residual signals at ~145 ppm correspond to <5% remaining OH-terminated PCHC.

Table S2: Depolymerization of $PCHC-O_2CCF_3$ using $LMg_2(OAc)_2$.[a]

Entry	Time / h	Conversion / %[b]	$M_{n,SEC}$ / g mol^{-1}[c]	Đ[d]
1	0	-	12,100	1.29
2	4	-	12,000	1.28
3	8	<5	12,100	1.24
4	24	<5	11,600	1.23

[a]Reaction conditions: $[PCHC-O_2CCF_3]_0$: $[Cat]_0$ = 300 : 1, $[PCHC]_0$ = 1 M in *p*-xylene ,120 °C.[b] Determined by ^{1}H NMR spectroscopy ($CDCl_3$) using the normalised integration of PCHC (δ = 4.65 ppm) against an internal standard of 1,3,5-trimethoxybenzene (δ = 6.01 ppm).[c] Calculated by SEC relative to polystyrene standards in THF eluent.[d] $Đ_M = M_w/M_n$.

Computational Modelling

Density Functional Theory calculations details

DFT calculations were performed using Gaussian16 suite of codes (revision A.03).[4] Geometries were fully optimized without symmetry or geometry constraints. Geometry optimization were carried out using the ωb97XD hybrid functional developed by Chai and Head-Gordon[6]. The 6-31+G(2d,p)[7] basis set was used for calculations. was used for the metal-free mechanism. Solvent effects in toluene were modelled using the cpcm model.[8]

Stationary points were verified as minima by the absence of imaginary frequencies following calculation of a vibrational frequency spectrum. Transition states were verified as such by the presence of a single imaginary frequency in the calculated vibrational frequency spectrum corresponding to the reaction coordinate.

Free enthalpies were calculated within the harmonic approximation for vibrational frequencies using Goodvibes software,[5] for a temperature of 393.15 K and catalytic species concentration of 0.003333 mol L^{-1}.

This research made use of the Balena High Performance Computing (HPC) Service at the University of Bath.

Full coordinates for all the stationary points, together with computed energies and vibrational frequency data, are available via the corresponding Gaussian16 output files and calculation spreadsheet, stored in the open-access digital repository DOI 10.6084/m9.figshare.17111591.

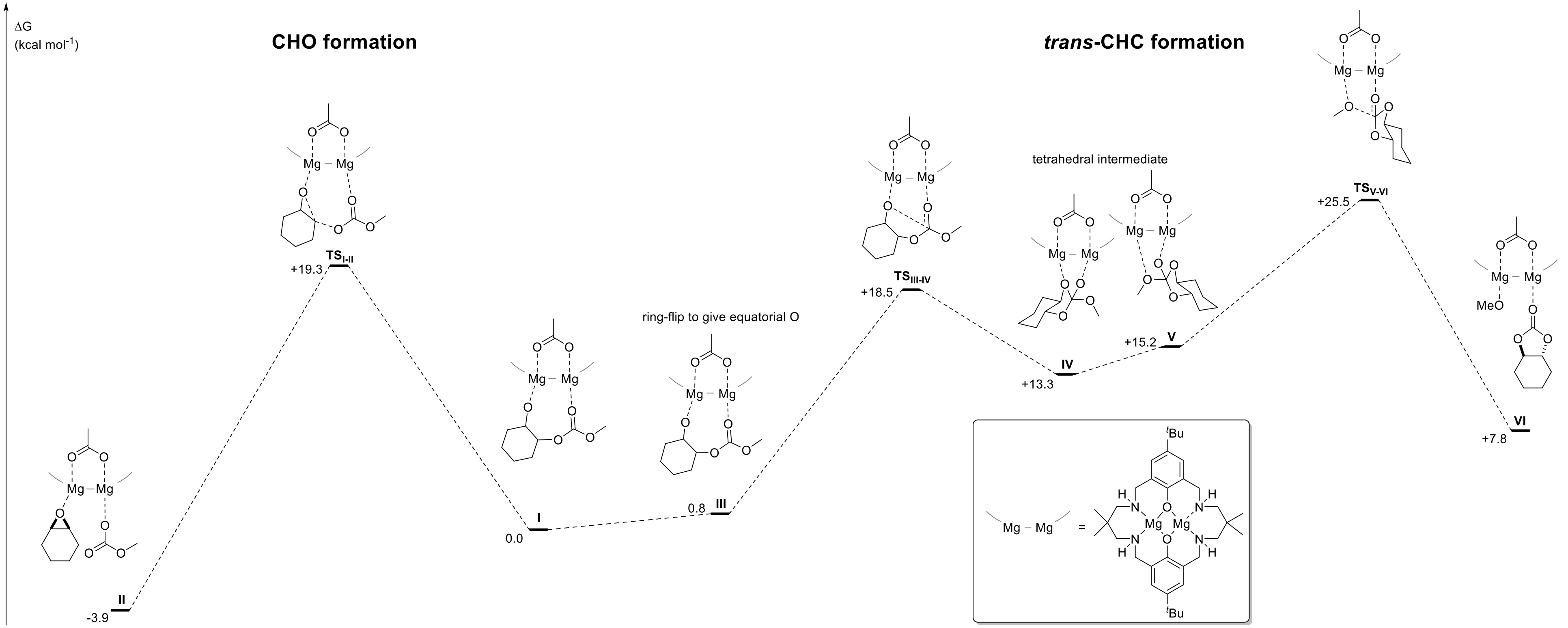

Figure S13: Computed free-enthalpy profile for the ring closing of PCHC to CHO and the backbiting to *trans*-CHC. Protocol: ωb97xD/ 6-31G+(2d,p)/cpcm = toluene/Goodvibes correction to 393.15 K and $[I]_0$ = 0.003333 M.

Table S3: Computed Free Gibbs Energy of structures given in Fig. S13 and their ΔΔG, relative to **I** (uncorrected and corrected with Goodvibes).

Entry	Structure	G (Hartree)[a]	ΔΔG (kcal mol^{-1})[a]	G (Hartree)[b]	ΔΔG (kcal mol^{-1})[b]
1	**I**	-2938.544496	0.0	–2938.583623	0.0
2	**TS$_{I-II}$**	-2938.513100	+19.7	–2938.552913	+19.3
3	**II**	-2938.550924	–4.0	–2938.589932	–3.9
4	**III**	-2938.543411	0.7	–2938.582413	+0.8
5	**TS$_{III-IV}$**	-2938.515111	+18.5	–2938.554171	+18.5
6	**IV**	-2938.524127	+12.8	–2938.562469	+13.3
7	**V**	-2938.521338	+14.6	–2938.559482	+15.2
8	**TS$_{V-VI}$**	-2938.503458	+25.8	–2938.543050	+25.5
9	**VI**	-2938.532152	+7.8	–2938.571238	+7.8

Calculations performed using rωb97xD functional, 6–31G+ (2d,p) basis set for all atoms and cpcm solvent model for toluene. [a]Temperature = 298.15 K, $[I]_0$ = 1.0 M. [b]Goodvibes correction applied at 393.15 K and $[I]_0$ = 0.003333 M.

Energy span calculations[9]

$$TOF = \frac{k_B T}{h} e^{-\Delta G/RT}$$

Formation of CHO:

ΔG = 19.3 kcal mol^{-1} = 80751 J mol^{-1}

$$TOF = \frac{1.381 \times 10^{-23} \times 393.15}{6.626 \times 10^{-34}} e^{\frac{-80751}{8.314 \times 393.15}} = 153\ s^{-1}$$

Formation of *trans*-CHC:

ΔG = 25.5 kcal mol^{-1} = 106692 J mol^{-1}

$$TOF = \frac{1.381 \times 10^{-23} \times 393.15}{6.626 \times 10^{-34}} e^{\frac{-106692}{8.314 \times 393.15}} = 0.0547\ s^{-1}$$

Ratio of CHO and *trans*-CHC TOFs:

$$\frac{153}{0.0547} = 2800$$

Theoretical selectivity:

$$\%CHO = \frac{153 - 0.0547}{153 + 0.0547} \times 100 = 99.9\ \%$$

References

1. a) M.R. Kember, P.D. Knight, P.T.R. Reung and C.K. Williams, *Angew. Chem. Int. Ed.*, **2009,** 48, 931-933. b) M.R. Kember and C.K. Williams, *J. Am. Chem. Soc.*, **2012**, 134, 15676-15679.
2. T. M. McGuire, E. M. López-Vidal, G. L. Gregory and A. Buchard, *J. CO_2 Util.*, **2018**, 27, 283-288.
3. Spyros, A.; Argyropoulos, D. S.; Marchessault, R. H. *Macromolecules*, **1997**, 30, 327-329.
4. Gaussian 16, Revision A.03, Frisch, M. J.; Trucks, G. W.; Schlegel, H. B.; Scuseria, G. E.; Robb, M. A.; Cheeseman, J. R.; Scalmani, G.; Barone, V.; Petersson, G. A.; Nakatsuji, H.; Li, X.; Caricato, M.; Marenich, A. V.; Bloino, J.; Janesko, B. G.; Gomperts, R.; Mennucci, B.; Hratchian, H. P.; Ortiz, J. V.; Izmaylov, A. F.; Sonnenberg, J. L.; Williams-Young, D.; Ding, F.; Lipparini, F.; Egidi, F.; Goings, J.; Peng, B.; Petrone, A.; Henderson, T.; Ranasinghe, D.; Zakrzewski, V. G.; Gao, J.; Rega, N.; Zheng, G.; Liang, W.; Hada, M.; Ehara, M.; Toyota, K.; Fukuda, R.; Hasegawa, J.; Ishida, M.; Nakajima, T.; Honda, Y.; Kitao, O.; Nakai, H.; Vreven, T.; Throssell, K.; Montgomery, J. A., Jr.; Peralta, J. E.; Ogliaro, F.; Bearpark, M. J.; Heyd, J. J.; Brothers, E. N.; Kudin, K. N.; Staroverov, V. N.; Keith, T. A.; Kobayashi, R.; Normand, J.; Raghavachari, K.; Rendell, A. P.; Burant, J. C.; Iyengar, S. S.; Tomasi, J.; Cossi, M.; Millam, J. M.; Klene, M.; Adamo, C.; Cammi, R.; Ochterski, J. W.; Martin, R. L.; Morokuma, K.; Farkas, O.; Foresman, J. B.; Fox, D. J. Gaussian, Inc., Wallingford CT, **2016**.
5. G. Luchini, J. V. Alegre-Requena, I. Funes-Ardoiz and R. S. Paton, *F1000Research*, **2020**, 9, 291.
6. a) J. Chai and M. Head-Gordon, *Phys. Chem. Chem. Phys.*, **2008**, 10 6615-6615. b) J. Chai and M. Head-Gordon, *Chem. Phys. Lett.,* **2008**, 467, 176-178.
7. a) R. Ditchfield, W. J. Hehre and J. A. Pople, *J. Chem. Phys.*, **1971**, 54, 724-728. b) W. J. Hehre, R. Ditchfield and J. A. Pople, *J. Chem. Phys.*, **1972**, 56, 2257-2261. c) P. C. Hariharan and J. A. Pople, *Theor. Chem. Acc.*, **1973**, 28, 213-222. d) P. C. Hariharan and J. A. Pople, *Mol. Phys.*, **1974**, 27, 209-214. e) M. S. Gordon, *Chem. Phys. Lett.*, **1976**, 76, 163-168. f) M. M. Francl, W. J. Pietro, W. J. Hehre, J. S. Binkley, D. J. DeFrees, J. A. Pople and M. S. Gordon, *J. Chem. Phys.*, **1982**, 77, 3654-3665. g) R. C. Binning Jr. and L. A. Curtiss, *J. Comp. Chem.*, **1990**, 11, 1206-1216. h) J.-P. Blaudeau, M. P. McGrath, L. A. Curtiss and L. Radom, *J. Chem. Phys.*, **1997**, 107, 5016-5021. i) V. A. Rassolov, J. A. Pople, M. A. Ratner and T. L. Windus, *J. Chem. Phys.*, **1998**, 109, 1223-1229. j) V. A. Rassolov, M. A. Ratner, J. A. Pople, P. C. Redfern and L. A. Curtiss, *J. Comp. Chem.*, **2001**, 22, 976-984.
8. a) R. Krishnan, J. S. Binkley, R. Seeger and J. A. Pople, *J. Chem. Phys.*, **1980**, 72 650-654. b) T. Clark, J. Chandrasekhar, G. W. Spitznagel and P. von R. Schleyer, *J. Comput. Chem.*, **1983**, 4, 294-301.
9. S. Kozuch and S. Shaik, *Acc. Chem. Res.*, **2011**, 44, 101-110.
